# Simian-Human Immunodeficiency Infection – Is the Course Set in the Acute Phase?

**DOI:** 10.1371/journal.pone.0017180

**Published:** 2011-02-17

**Authors:** Janka Petravic, Miles P. Davenport

**Affiliations:** Complex Systems in Biology Group, Centre for Vascular Research, University of New South Wales, Sydney, Australia; Karolinska Institutet, Sweden

## Abstract

Identifying early predictors of infection outcome is important for the clinical management of HIV infection, and both viral load and CD4+ T cell level have been found to be useful predictors of subsequent disease progression. Very high viral load or extensively depleted CD4+ T cells in the acute phase often result in failure of immune control, and a fast progression to AIDS. It is usually assumed that extensive loss of CD4+ T cells in the acute phase of HIV infection prevents the establishment of robust T cell help required for virus control in the chronic phase. We tested this hypothesis using viral load and CD4+ T cell number of SHIV-infected rhesus macaques. In acute infection, the lowest level of CD4+ T cells was a good predictor of later survival; animals having less than 3.3% of baseline CD4+ T cells progressed to severe disease, while animals with more than 3.3% of baseline CD4+ T cells experienced CD4+ T cell recovery. However, it is unclear if the disease progression was caused by early depletion, or was simply a result of a higher susceptibility of an animal to infection. We derived a simple relationship between the expected number of CD4+ T cells in the acute and chronic phases for a constant level of host susceptibility or resistance. We found that in most cases, the depletion of CD4+ T cells in chronic infection was consistent with the prediction from the acute CD4+ T cell loss. However, the animals with less than 3.3% of baseline CD4 T cells in the acute phase were approximately 20% more depleted late in the infection than expected based on constant level of virus control. This suggests that severe acute CD4 depletion indeed impairs the immune response.

## Introduction

The disease course in untreated human immunodeficiency virus (HIV) infection consists of an early acute phase characterized by extremely high viral loads and depletion of CD4+ T cells, followed by a largely asymptomatic chronic phase with more moderate viral loads and a slow loss of CD4+ T cell pool after partial recovery, and finally emergence of immunodeficiency, opportunistic infections and death. A similar but more rapid course of infection is seen in some non-human primates models of HIV, using infections with simian and simian-human immunodeficiency viruses (SIV and SHIV respectively).

Despite the differences in the disease course in the three types of untreated infections, prolonged survival in HIV-1 [1], SIV_mac_ [2] and SHIV [3] was found to be linked to better viral control and CD4+ T cell recovery during chronic phase. This is commonly explained by the fact that CD4+ T cells play an important role in immune control, providing help for both antibodies and CD8+ T cells responses, which act to control infection. This is in agreement with studies in mice where the absence of CD4+ T cells in primary infection limits the subsequent ability of CD8+ T cells to respond to secondary infection [1], [4], [5], [6], [7]. Thus, the development of AIDS may occur when the density of CD4+ T cells drops below a limit necessary to provide help (the threshold being around 200 cells/µL of blood for HIV infection), leading to functional defects in CD8+ T cells and antibody-producing B cells.

Experiments using SHIV infection in rhesus macaques indicate that the difference between immunodeficiency and prolonged survival may be programmed early – the outcome could be traced back to the degree of severity of viremia and CD4+ T cell loss during the acute phase [1], [2], [3], [8], [9]. While a sustained better immune response during the whole course of disease would produce better viral control and CD4+ T cell preservation in all phases of infection, an extremely severe acute phase could in principle cause some additional irreversible damage to the immune system, further compromising the long-term outcome. Some indications for this effect come from SHIV challenge of rhesus macaques. First, the degree of partial recovery of CD4+ T cells after the acute phase seems to decrease as depletion in the acute phase increases [3]. If the nadir in CD4+ T cells in acute phase drops below approximately 20 cells/µL, there is no observed partial recovery, and the animals experience a continued decline in CD4+ T cell numbers and an increase in viral load [8]. In addition, the disease outcome can be modulated by early interventions that lower the acute viremia and preserve CD4+ T cells at nadir, such as early passive administration of neutralizing antibodies [10], [11], early initiation of short-term antiretroviral treatment [3] and vaccination [12], [13]. This suggests that there might exist a threshold in the severity of the acute disease, above which later virus control is impaired. Very high peak viral loads or a extremely low CD4+ T cell nadir [14] may lead to inflammation and loss of lymph node architecture. Similarly, severe early or prolonged loss of the CD4+ T cells in the gut mucosal barrier can result in microbial translocation, which could cause generalized T cell activation and tissue damage [15].

Although early and acute high viral load and severe CD4+ T cell depletion are associated with a poor long-term outcome, it is not clear that this is causative: if animals vary in their susceptibility to infection, then highly susceptible animals would be expected to have highly depleted CD4+ T cells and high viral loads in both the acute and chronic phases of infection. Conversely, relatively resistant animals would have a low viral load and well-preserved CD4+ T cells in the acute and chronic phase. In this case, high early viral load and severe CD4+ T cell depletion are associated with a high late viral load and poor outcome, but the association is not causative. Alternatively, a high early viral load and extensive CD4 depletion may reduce CD4+ T cell help, and compromise later immune control, which in this case would be causative.

In this paper, we explore the early predictors of long-term outcome in SHIV_89.6P_ infection. CXCR4-tropic SHIV_89.6P_ infection causes an accelerated disease progress with rapid systemic loss of all CD4+ T cell phenotypes compared to CCR5-tropic SIV and HIV. It has been used as a model for studies of potential protective ability of vaccines against HIV-1 because it permits an early assessment of vaccine efficacy. Despite their higher pathogenicity, X4-tropic SHIVs have proven to be easier to control by vaccination than their more moderate CCR5-tropic counterparts SIV_mac_ and HIV [16], [17]. This infection is particularly suitable for our study because SHIV_89.6P_ infects both naïve and memory CD4+ T cells, and the loss of CD4+ T cells measured in blood can be taken to approximately represent the overall loss of CD4+ T cells in all anatomical compartments.

We first show that there is a threshold level of preservation of CD4+ T cells and a threshold viral load in the acute phase that correspond to the chronic phase thresholds of prolonged survival (for longer than 600 days) in CXCR4-tropic SHIV_89.6P_ infection in rhesus macaques. We then use a modeling approach to show that a simple theoretical relationship should exist between CD4 T cell levels in acute and chronic infection under the conditions of constant immune control during the whole course of disease. However, if early CD4+ T cell depletion leads to impaired immunity, then we would expect CD4 depletion in the chronic phase of infection to be higher than predicted by this theoretical relationship.

Our results show that there is indeed a well-defined threshold level of remaining CD4 T cells in the acute phase that corresponds to survival (and a corresponding somewhat less well-defined limiting viral peak). Interestingly, animals that experience CD4 preservation above this threshold show the predicted relationship between acute and chronic CD4+ T cell levels, suggesting immune control is maintained. By contrast, animals with the fraction of remaining CD4+ T cells in the acute phase lower than this threshold also show significantly less than predicted remaining CD4+ T cells in the chronic phase. This suggests that the threshold level of CD4+ T cells predicting survival corresponds to the minimum level of CD4+ T cells required to preserve immune function in the chronic phase of infection.

## Results

### Predicting survival based on viral load and CD4+ T cell number

**Figure 1:**
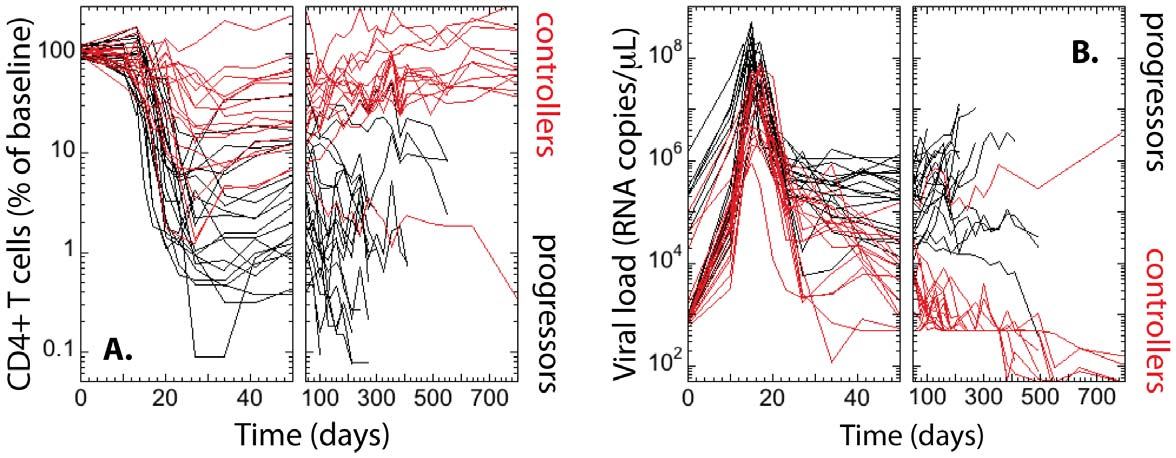
Measures of disease progress in SHIV-infected rhesus macaques. The animals are divided into controllers (red) that survived longer than 600 days, and progressors (black) that were euthanized earlier due to severity of symptoms. (A) Longitudinal data for the CD4 T cells (in % of baseline) and (B) viral load.

In Figure 1 we show the longitudinal data for CD4+ T cell count in peripheral blood compared to baseline (A) and plasma viral load (B) for the 35 monkeys from the Shiver *et al.* study [13]. The baseline CD4 count was determined for each animal as the average value of two pre-infection time points. We divided all monkeys into two groups – those that survived for longer than 600 days (16 monkeys that we call “controllers”), and those that died or had to be euthanized earlier (19 monkeys that we call “progressors”). Out of 21 vaccinated animals, 15 controlled the infection and 6 were progressors (five DNA-based vaccines and one MVA), while only one of 14 unvaccinated animals controlled the infection. The timelines for the controllers are shown in red, while the black lines represent the progressors. Progressors had distinctly lower levels of CD4+ T cells at steady state and in the acute phase than controllers, less CD4 recovery after the acute phase than controllers, and higher viral set point and peak.

We first used diagnostic tests to determine how well our classification of animals into controllers and progressors could be reproduced on the basis of the fraction of remaining CD4 T cells in the chronic phase and set point viral load instead of survival. We were looking for the threshold values of the chronic CD4+ T cell preserved fraction *T* ^*^/*T*_0_ and set point viral load that best reproduced the division into progressors and controllers based on survival longer than 600 days.

**Figure 2:**
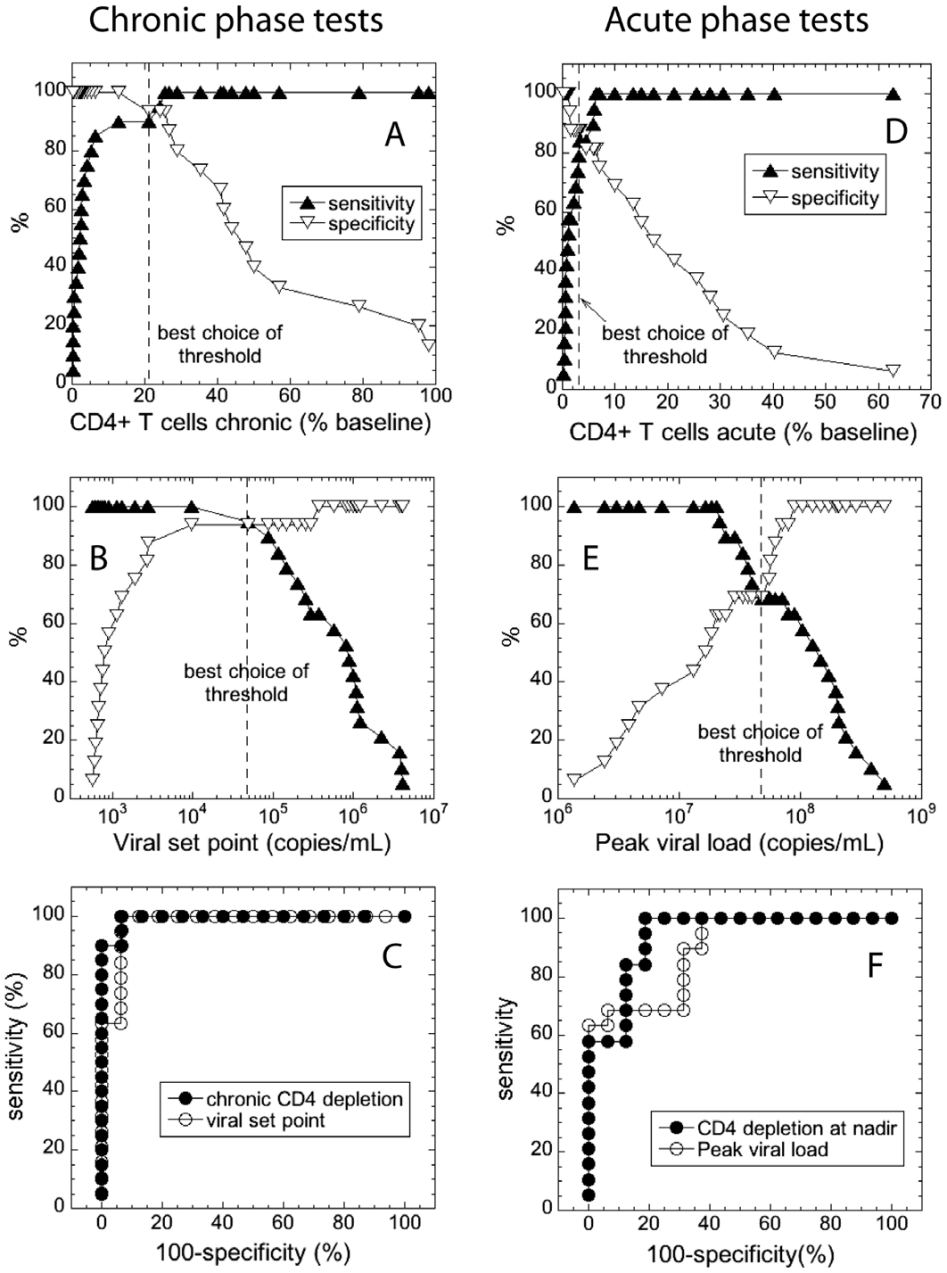
Measures of accuracy of the survival tests based on CD4 preservation and viral load in the chronic and acute phases of SHIV infection. (A–C) Chronic phase tests: (A) sensitivity and specificity of the test based on chronic level of CD4+ T cells; (B) sensitivity and specificity of the test based on set point viral load, vertical dashed line represents the best choice of threshold; (C) ROC areas of the chronic phase tests; (D–E) Acute phase tests: (D) sensitivity and specificity of the test based on acute level of CD4+ T cells; (E) sensitivity and specificity of the test based on peak viral load, (F) ROC areas of the acute phase tests.

We considered progressors “positive” for early death and controllers “negative”. If we tested for early death using the chronic depletion of CD4+ T cells, for each choice of the threshold we would have a number of “true positives” (progressors that test positive), “false positives” (controllers that test positive), true negatives (controllers that test negative) and false negatives (progressors that test negative). We chose the best threshold value as the one that simultaneously maximized sensitivity, i.e. the fraction of true positives out of all animals that test positive (Eq.1), and the specificity, i.e. the fraction of true negatives out of all animals that test negative (Eq.2). The summary of the measures of accuracy of the two diagnostic tests is shown in Figure 2A-C (sensitivity – full triangles; specificity – open inverted triangles) and in Tables 1 and 2. The best classification was obtained with the threshold chronic CD4 preservation of 24.1% and viral load of 4.83×10^4^ RNA copies/mL (dashed vertical lines in Figure 1A and B) when values of sensitivity and specificity were the closest. The overall accuracy of the tests based on chronic CD4 depletion or viral load is compared in Figure 1C. A more accurate test would be the one for which the area under the curve of sensitivity as a function of (1-specificity), or the so-called “ROC area”, is closer to unity. Tests based on both chronic phase quantities had ROC areas higher than 0.95, with CD4 preservation at 0.993 ranking a little better than viral load at 0.977 (only one animal wrongly classified at the best threshold of CD4 preservation, compared to two for viral set point). The ROC areas for the two chronic phase measures were not significantly different (*p* = 0.459)

**Table 1:** Summary of measures of accuracy of survival tests based on depletion of CD4+ T cells.

Test	CD4+ T cells chronic phase (% of baseline)	CD4+ T cells acute phase (% of baseline)
ROC area (standard error)	0.993 (±0.008953)	0.938 (±0.03299)
Best choice of threshold	75.9%	96.7%
Sensitivity	95.0%	84.2%
Specificity	93.3%	87.5%

**Table 2:** Summary of measures of accuracy of survival tests based on viral load.

Test	Set point viral load (RNA copies/mL)	Peak viral load (RNA copies/mL)
ROC area (standard error)	0.977 (±0.02428)	0.891 (±0.05304)
Best choice of threshold	4.83×10^4^	4.79×10^7^
Sensitivity	94.7%	84.2%
Specificity	93.4%	87.5%

Next, we quantified how well we could separate controllers from progressors on the basis of the acute phase measures. In Figure 2D and E we plotted the sensitivity and specificity of the tests based on CD4+ T cell levels in the acute phase (measured as % of baseline) and on viral load, respectively. The best combination of sensitivity and specificity was obtained for the threshold level of CD4+ T cells in the acute phase at 3.3% of baseline, and the threshold peak viral load of 4.79×10^7^ RNA copies/mL (Tables 1 and 2). Specificity and sensitivity at the best value of threshold were considerably higher for the test based on CD4 preservation (∼94%) than for the test based on viral load (∼86%), indicating that the test based on CD4 preservation is more accurate. This is confirmed by the larger ROC area of the acute CD4-based test (0.938, compared to 0.891 for the viral peak). The ROC area of the test based on the CD4+ T cells remaining in the acute phase was not significantly different from the area of the test based on the CD4+ T cells in the chronic phase (*p* = 0.144). However, the ROC area of the test based on peak viral load was significantly lower (*p* = 0.0400) than the area of the test based on the CD4+ T cells in the acute phase.

As expected, ROC areas and sensitivities and specificities at the best threshold were higher for the tests based on chronic phase, showing that the chronic phase is generally a better predictor of survival. However, CD4 preservation was a better predictor of survival than viral load in the acute phase.

### Insights from modeling into the importance of CD4+ T cells as a predictor

From the standard model of viral dynamics (Eq.3–5) we can get some insights into the reasons why preservation of CD4+ T cells is a better indicator of survival than viral load, although viral load and CD4 levels are strongly (negatively) correlated. According to the model, viral loads in the acute and chronic phases of infection depend on baseline CD4 number and virus-dependent parameters, such as virus infectivity, death rate of infected cells, virus production by infected cells and free virus clearance, all of which can be differently modified by individual host immune responses. On the other hand, the fractions of remaining CD4+ T cells in the acute (Eq.9) and chronic phases (Eq.10) only depend on the basic reproductive ratio (Eq.6), i.e. a single number that is a combination of all these individually varying parameters. This means that, with a constant level of total immune response (model parameters not depending on time) during the whole course of disease, the CD4 preservation in the acute phase would completely determine the final fraction of remaining cells in the steady-state. However, the peak viral load (Eq.7) depends on different individually varying parameters than the viral set point (Eq.8). This means that two animals with the same peak viral load can have different viral set points and different steady-state CD4+ T cell levels, and therefore different long term outcomes, even for the simple dynamics with constant total immune response described by the model. For example, one animal can have a much higher death rate of infected cells *δ* than the other (having better CD8+ T cell response), which may be compensated by a somewhat higher infectivity *β*. Its basic reproductive ratio *R*_0_ (Eq.6) would then still be lower than in the other animal, although both animals would have the same peak viral loads (Eq.7). However, the animal with the lower basic reproductive ratio would have higher acute and chronic CD4 levels and lower set point viral load than the animal with higher basic reproductive ratio. On the other hand, two animals having the same CD4 nadir will always have the same chronic CD4 level, irrespective of the specific parameters causing CD4 depletion.

### Is there irreversible damage to the immune system in the acute phase?

We have shown that the prolonged low level of CD4+ T cells in the chronic phase below the threshold of 24.1% of baseline leads to early death, and that this low level can be predicted by the CD4+ T cell level in the acute phase below 3.3% of baseline. What is not clear is if the relationship between the levels of CD4+ T cell preservation in acute and chronic infection is just the result of the intrinsic susceptibility of the animal (including the strength of the immune response), which determines both acute and chronic CD4+ T cell levels. Alternatively, if early CD4+ T cell depletion compromised immune control and drove later disease progression, then we expect that the level of CD4+ T cells in the chronic phase should be worse than expected from a simple model based on constant immune control. Thus, we need to first estimate the expected relationship between the acute and chronic phases of infection, and then observe if the latter is worse than expected.

**Figure 3:**
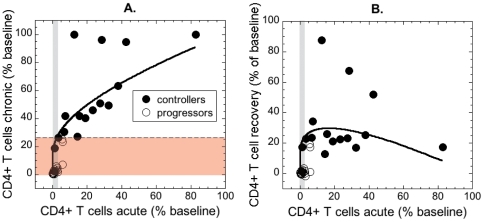
CD4+ T cells remaining in the chronic phase (as % of baseline) and their recovery as functions of the CD4+ T cells remaining at nadir. Full line represents the relationship expected for constant level of immune control. Grey area is the level of CD4+ T cells at nadir lower than the acute survival threshold of 3.3% of baseline. (A) Survival threshold of 24.1% of baseline based on chronic phase CD4 T cell level (dashed line bordering the red area) almost exactly corresponds to the acute threshold, based on the assumption of constant immune response. The animals with data points below the full line have less CD4+ T cells remaining in the chronic phase than expected from the nadir. (B) CD4+ recovery is defined as the difference between the nadir and the chronic level. The points above the curve represent better than expected recovery. All animals with the level of remaining CD4+ T cells lower than the survival threshold recover considerably less than expected.

We can make this estimate using the standard model of viral dynamics (Eq.3–5). If we plot CD4+ T cell level in the chronic phase *T* ^*^/*T*_0_ as a function of the fraction remaining in the acute phase *T*_min_/*T*_0_, we obtain the universal curve shown as solid black line in Figure 3A. Full circles represent the data for the controllers, and open circles represent the progressors. The grey area encloses CD4+ T cell levels in the acute phase lower than the survival threshold, as determined by the ROC analysis. The pink area is the fraction of remaining CD4 T cells in the chronic phase corresponding to the acute levels in the grey interval, as predicted by the model, if we assume constant immune response during the whole course of disease. It is immediately obvious that, for very low fractions of remaining CD4+ T cells in the acute phase, small improvement in CD4 preservation results in much larger improvement in the long-term outcome.

The horizontal dashed line in Figure 3A is the survival threshold for the chronic preservation of 24.1% of CD4+ T cells from the diagnostic test. If the immune response remained constant, 3.3% remaining CD4+ T cells in the acute phase would result in 27% remaining CD4+ T cells in the chronic phase, very close to the test threshold of 24.1%. Using the chronic survival threshold predicted by the model would result in only one more controller animal being wrongly classified as progressor based on the remaining CD4+ T cells in the chronic phase. The similarity between the theoretical and experimental chronic preservation thresholds corresponding to the preservation of 3.3% CD4+ T cells in the acute phase suggests that the chronic preservation above the survival threshold is a consequence of normal disease progression from the acute to the chronic phase, with constant levels of immune response differing among animals.

The standard model can also be used to estimate the amount of CD4+ T cell recovery between the CD4 the acute and the chronic phase, depending on the acute depletion, that we would expect to see if the immune response were constant in time. The full black line in Figure 3B shows this dependence. The amount of recovery is presented as percent of baseline CD4+ T cell level (*T* ^*^-*T*_min_)/*T*_0_. The amount of expected CD4 recovery increases rapidly with increasing acute CD4+ T cell level, up to approximately 15% preservation, and is very low when acute CD4+ T cells are almost completely depleted. Therefore the lack of CD4+ T cell recovery observed after extremely low acute levels does not need to be a consequence of irreversible damage to the immune system, but may be a normal progress in the case of a very susceptible animal.

**Figure 4:**
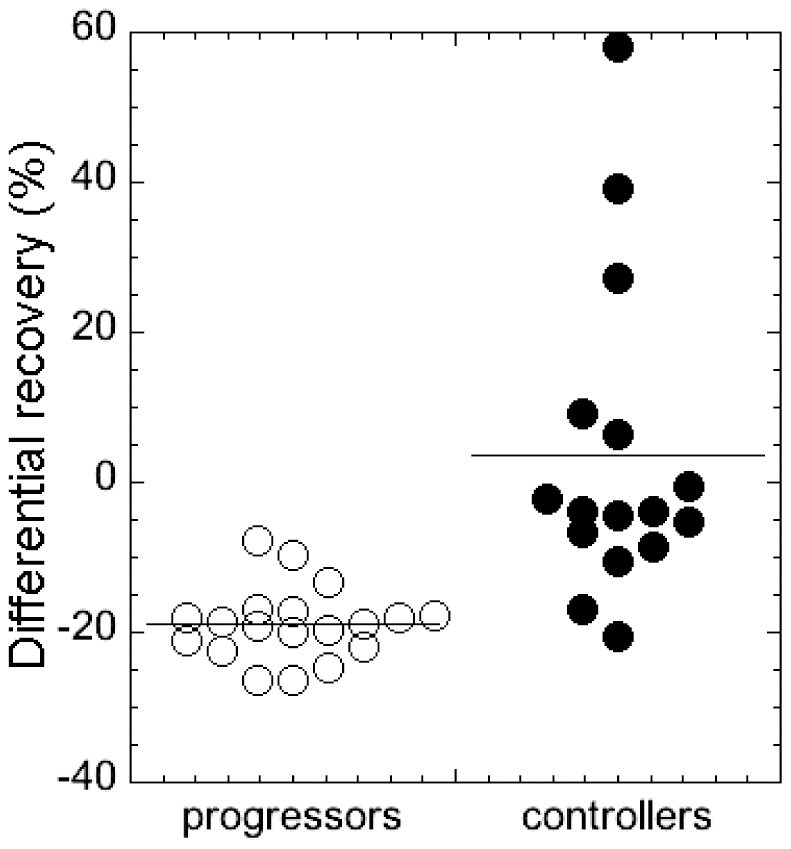
Differential recovery in progressors and controllers. We defined the “differential recovery” as the difference between the actual recovery and the amount of recovery expected if the immune response were constant. The differential recovery in progressors was significantly lower than in controllers (Mann-Whitney *p*<0.0001).

When the data points appear below the theoretical curves in Figures 3A or 3B, this means that the animals had fewer CD4+ T cells in the chronic phase than expected from the acute phase. We can see that most animals (with the exception of five controllers) do worse than expected in the long run – most points are below the theoretical curves in Figure 3A and 3B. In order to quantify how much less the animals recover on average after the acute CD4 loss, we calculate the “differential recovery” – the difference between the actual recovery (*T* ^*^-*T*_min_)/*T*_0_ and the amount of recovery expected if the immune response were constant (as estimated from the model). We obtain the overall median differential recovery of −16.9%, meaning that over all, the animals do worse than expected by 16.9%. Progressors do worse than expected by (median) 18.8%, while controllers do worse by (median) 3.9%, which is significantly better than progressors (Figure 4, Mann-Whitney p<0.0001).

**Figure 5:**
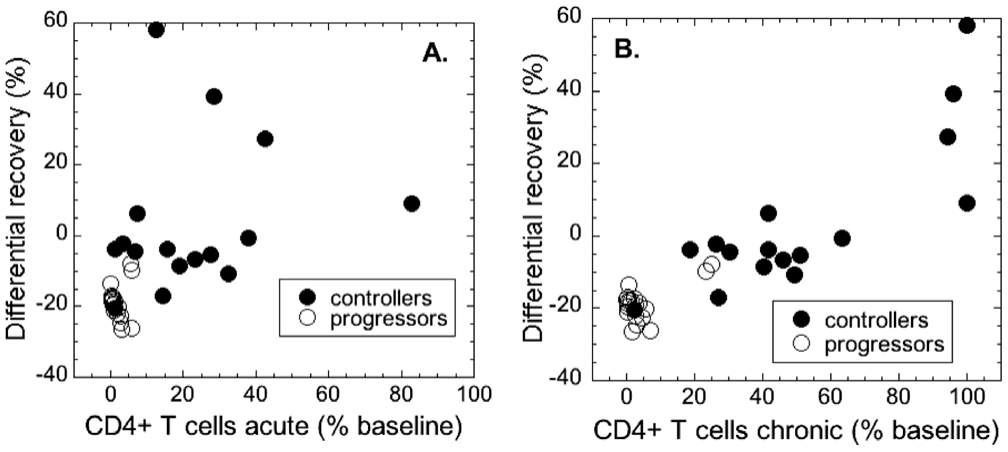
Differential recovery is significantly positively correlated to CD4+ T cell preservation: (A) in the acute phase (Spearman *r* = 0.536, *p* = 0.0009); (B) in the chronic phase (Spearman *r* = 0.745, *p*<0.0001). Grey rectangles represent CD4+ T cell levels below the survival thresholds. If we consider only the points with CD4+ T cells above the survival thresholds (outside the grey rectangles), (A) in the acute phase the correlation is no more significant (Spearman *r* = 0.346, *p* = 0.1743), but (B) it is still significant in the chronic phase (Spearman *r* = 0.681, *p* = 0.0052)

The progressors, who have lower levels of CD4+ T cells both in acute and in chronic phase, recover less than expected in the chronic phase. This suggests that weaker recovery might be associated with the level of early CD4+ T cell depletion. In Figure 5 we plot the differential recovery against the acute CD4 level (Figure 5A) and CD4 level in the chronic phase (Figure 5B). In both cases there is a significant over-all positive correlation (Spearman *r* = 0.536, *p* = 0.0009 in the acute phase and Spearman *r* = 0.745, *p*<0.0001 in the chronic phase). However, the significance of the correlation of recovery with the acute CD4 level in Figure 5A heavily relies on the cluster of points at very low CD4 fraction belonging to progressors. If we remove this cluster of points with the acute CD4+ T cell level below 3.3% of baseline (the acute survival threshold in the grey rectangle) the positive correlation loses significance (Spearman *r* = 0.346, *p* = 0.1743). This may indicate that either there is indeed some damage caused by the loss of more than 96.7% CD4+ T cells in the acute phase, but that preservation of more than 3.3% CD4+ T cells might be sufficient for the maintenance of stable immune function.

On the other hand, if we remove the points below the chronic survival threshold of 24.1% (the grey rectangle) from Figure 5B, the significant positive correlation remains (Spearman *r* = 0.681, *p* = 0.0052), which is expected because the chronic CD4+ T cell level is the result of CD4 recovery after acute depletion.

## Discussion

Better preservation of CD4+ T cells in the acute phase of SHIV and SIV infection of rhesus macaques has been observed to lead to better CD4 recovery and improved outcome of the disease later on [2], [3], even when the acute CD4 preservation was a consequence of early interventions. Analysis of a data set of 35 SHIV-infected rhesus macaques showed the expected result that long-term survival is associated with set point viral loads below 4.83×10^4^ and the chronic phase CD4+ T cell levels above 24.1% of baseline. In this paper we addressed two questions: is there a similarly well defined survival threshold for CD4+ T cell depletion and viral load in the acute phase, and if there is, is it associated with a decline in immune response later.

It was indeed possible to find a well defined survival threshold in the acute phase: if CD4+ T cells in the acute phase drop below 3.3% of baseline, this loss is associated with almost no CD4 recovery in the chronic phase, and early death (in less than 600 days).

We used modeling to predict the hypothetical chronic phase CD4+ T cell loss based on the CD4+ T cell level in the acute phase assuming constant level of virus control between acute and chronic phase. Constant virus control in this context would mean the combined effect of intrinsic susceptibility of individual animals at cellular level (e.g. ability to produce cytoplasmic defence proteins) and all types of immune responses including innate and adaptive immune mechanisms. While the relative importance of these effects could shift during the disease course, their combined effect on the control of viral replication may still be constant.

Modeling revealed two main features of the disease progress between the acute and the chronic phase, expected with unchanged immune control. Firstly, if the cells targeted by virus become highly depleted (more than 95%) in the acute phase, a small improvement in virus control would lead to a small increase in their numbers in the acute phase, and a comparatively much larger increase in the chronic phase. Similar pattern was observed in SIV-infected rhesus macaques, where the variation in the intrinsic ability of their CD4+ T cells to produce TRIM5α had greater effect in late than in early infection [18].

Secondly, if we define CD4 recovery as the difference between the CD4 count in the acute and chronic phase, then we find that we can expect the recovery to decrease with the decrease in remaining CD4+ T cells at nadir even without any deterioration in immune control. In other words, lower CD4 recovery in the chronic phase of viremia in animals with severe acute CD4 depletion could be just a consequence of a constant high level of susceptibility in these animals.

We compared the observed CD4 recovery in the chronic phase to the expected recovery estimated from the level of CD4 preservation at nadir assuming constant level of virus control. We found that recovery was significantly less than expected in the animals with nadir CD4 preservation below the survival threshold. In addition, we observed that the difference between observed and expected recovery was over-all significantly positively correlated with CD4 preservation in the acute phase, but that the correlation lost significance when we removed the animals with the acute CD4+ T cell level lower than the survival threshold. These observations are consistent with a model in which there is a threshold level of acute CD4+ T cells (3.3% of baseline), below which there is a decline in immune function and a tendency for rapid progression. Possible reasons could be insufficient CD4 help necessary for sustained immune control, or early lymphoid or mucosal tissue damage [19] due to inflammatory processes associated with low CD4+ T cell levels. In contrast, when CD4+ T cells in acute phase were preserved above the threshold, the total effect of immune control did not seem to decline, despite the virus-specific CD8+ T cells peaking in acute phase and subsiding later. In addition, the majority of controller animals with less than threshold acute depletion were vaccinated, suggesting that in SHIV infection the early preservation of T cells caused by vaccination can make lasting immune control possible.

In CCR5-tropic SIV and HIV infections, total body CD4+ T cell loss in the primary phase is almost never as extreme as 97%, so that it should not compromise later antiviral responses. This suggests that the acute loss of CD4+ T cells is unlikely to contribute to the late decline in virus control in HIV, which may be caused by other complications occurring later in the disease course. However, many studies have shown that the loss and qualitative impairment of HIV-specific T-cell help occurs very early in infection and cannot therefore be predicted by the overall numbers of circulating CD4+ T-cells.

## Materials and Methods

### Experimental data

In a previously published study [13], 35 rhesus macaques (*Macaca mulatta*) were challenged intravenously with 50% monkey infectious doses of CXCR4-tropic SHIV_89.6P_. 14 animals in this group were unvaccinated controls, while 21 were vaccinated with a variety of regimens, consisting of SIV gag-containing plasmid DNA (with different adjuvants), modified vaccinia Ankara, and adenovirus type 5 vectors, as previously reported [13]. The vaccinated animals were challenged at 6 weeks or at 12 weeks after the final boost. Viral loads and CD4+ T-cell counts were monitored in peripheral blood every 2 to 4 days until 4 weeks after infection and then weekly.

### Data analysis

Initially, we divided the animals into progressors, which died before day 600 of infection, and controllers, which survived beyond 600 days. We designed diagnostic tests based on CD4+ T cell level and on viral load in order to predict early death, i.e. we found the threshold CD4+ T cell level and threshold viral load in acute and chronic phase of infection that best corresponded to the classification into progressors and controllers. As the acute phase measures we chose the CD4+ T cell level at nadir and the peak viral load. In the chronic phase we used the set point CD4+ T cell level and viral load, which we defined as the averages between day 100 and day 200 of infection, or between day 100 and death if the animal died before day 200. All animals survived longer than 100 days.

For every recorded value of a particular measure (CD4+ T cell level or viral load) chosen as a threshold, we found the number of animals correctly or wrongly classified as progressors (true positives TP or false positives FP respectively), and correctly or wrongly classified as controllers (true negatives TN or false negatives FN). The accuracy of a particular classification is measured in terms of specificity and sensitivity [20]. The sensitivity of the test for a given choice of threshold is the fraction of all progressors (TP+FN) that are correctly classified as progressors (TP); and the specificity is the fraction of all controllers (TN+FP) that are correctly classified as controllers (TN),
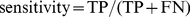
(1)

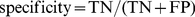
(2)


For an ideal indicator of survival, there would exist a threshold (or a threshold interval) for which all progressors and controllers classified correctly (FP = FN = 0), i.e. sensitivity and specificity would both be equal to unity for the best choice of threshold, while one of them would be less than unity for values of threshold outside the correct range. This is not the case for any of the measures that we have chosen. We defined the best choice of threshold as the one that maximizes both specificity and sensitivity, i.e. for which sensitivity and specificity had the closest values.

Sensitivity and specificity can be used to assess a classification based on the chosen threshold value of the test quantity (in our case CD4 depletion or viral load). Sensitivity and specificity for the best choice of threshold indicate how accurate is a test based on a particular quantity (how well defined is the threshold value). A more rigorous way to evaluate the overall accuracy of a test based upon a particular measure is to plot sensitivity as a function of (1-specificity) for all recorded values of the test quantity and find the area under the curve (the so-called receiver-operator characteristic (ROC) area [21]). If controllers tend to test higher, ROC area represents the probability that the test value for a randomly chosen controller is higher than the test value of a randomly chosen progressor (and opposite for tests for which controllers test lower). If a perfectly clear-cut threshold value separated controllers and progressors, the ROC area would be equal to unity. Larger ROC area indicates that a test based on a particular quantity is more accurate. We compared the ROC areas, obtained using different quantities as the basis of classification on the same data set, using the method described in [22].

Data analysis was performed using Graphpad Prism v.5.0a for Mac OSX.

### Model

The standard model [23], [24], [25], [26] of viral dynamics has been used to study HIV infection for a number of years [27]. We have shown that it describes well the dynamics of viral load and CD4+ T cells in the acute phase of SHIV_89.6P_ infection [28], [29], where we have access to both viral load and target cell number.

The standard model of virus dynamics,
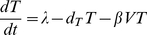
(3)

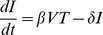
(4)

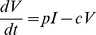
(5)describes the relationships between the change in the number of uninfected cells *T* that are targets for the virus, infected cells *I* and free virus particles *V* in a given volume of blood or tissue. The parameters *λ*, the production rate of new targets, and *d_T_*, the loss rate of target cells, describe the disease-free target cell dynamics. The disease-free equilibrium number of target cells is equal to *T*_0_ = *λ*/*δ_T_*. The infectivity *β* characterizes the probability of a virus particle infecting a target cell, and *δ* is the death rate of infected cells, *δ*≫*d_T_*. Free virus is produced by infected cells at the rate *p* and is cleared at the rate *c*. In this model, different types of immune response are assumed to modify the virus parameters *δ*, *c*, *β* and *p*. All the parameters (and consequently the immune response) are assumed to be constant in time.

In this simple model, the course of the infection is largely determined by the basic reproductive ratio of the virus *R*_0_. It is defined as the number of infected cells generated initially by one infected cell in its lifetime, and as such is a measure of viral replication. In terms of the parameters for this model it is
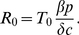
(6)


The infection can spread only if *R*_0_>1. In this case, viral load generally grows exponentially to the peak *V_P_*, which for the parameter range characteristic for SHIV infection can be approximated by [24], [28]

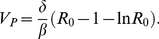
(7)


After reaching the peak, viral load decays and finally reaches the set point value V^*^,
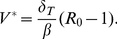
(8)


Peak viral load and viral set point are positively correlated to the basic reproductive ratio, but in addition depend separately on different viral parameters.

Target cells drop to the nadir *T*_min_, then partly recover, and settle at the steady-state value T^*^. The nadir of target cells in the standard model applied to SHIV is the solution of the equation [28]

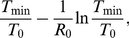
(9)and the set point number is
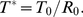
(10)


The fractions of target cells remaining at nadir *T*_min_/*T*_0_ and at the steady state *T* ^*^/*T*_0_ only depend on the basic reproductive ratio *R*_0_. This means that the dependence of the preserved steady state target cell fraction on the remaining fraction at nadir can be represented as a single universal curve parametrized by *R*_0_. Note that there is no fitting required for this curve – for every value of *R*_0_>1 there is only one value for the preservation of target cells *T*_min_/*T*_0_ at nadir and only one corresponding value for the preservation *T* ^*^/*T*_0_ at the steady state. The relationship between the remaining nadir and chronic fractions is universal for all viral infections for which the basic model applies, provided that the uninfected target cell replacement and loss rates are small, and that the virus clearance rate is much higher than the death rate of infected cells (the conditions under which the expressions Eq.7-9 were derived [28]). The important limitation is that this relationship can only be useful when the target cell abundance can be measured directly. For this reason the model is most easily applied to the infection with CXCR4-tropic virus.
